# (1*E*,2*E*)-1,2-Bis[(1-benzyloxymethyl-1*H*-indol-3-yl)methylidene]hydrazine

**DOI:** 10.1107/S1600536812042493

**Published:** 2012-10-20

**Authors:** Jacob A. Myers, Frank R. Fronczek, Steven F. Watkins

**Affiliations:** a Department of Chemistry, Louisiana State University, Baton Rouge, LA 70803-1804, USA

## Abstract

The title compound, C_34_H_30_N_4_O_2_, lies on an inversion center and consists of two 3-substituted-1*H*-indole units linked by a 1,2-dimethyl­enehydrazine bridge. It is one of numerous examples in which two aromatic ring systems are joined by this 4-atom bridge. The geometry of the centrosymmetric bridge is: C(arom)—C = 1.444 (3), C=N = 1.284 (3), N—N = 1.414 (4) Å, C(arom)—C=N = 122.6 (2) and C=N—N = 111.9 (2)°. The nine non-H atoms of the indole unit lie in a plane (δ_r.m.s._ = 0.0089 Å) which is twisted 6.0 (2)° with respect to the hydrazine bridge plane. The benzyl­oxymethyl substituents do not lie in the plane of the rest of the mol­ecule and are in a folded rather than an extended conformation. This is described by the three torsion angles in the middle of the C=N—C—O—C_Bz_ group, *viz.* 98.5 (3), −62.1 (3), and −66.3 (2)°.

## Related literature

For the synthesis, see: Shui (1994 ▶). For related structures, see: Burke-Laing & Laing (1976 ▶); Mom & de With (1978 ▶); Biswas *et al.* (1999 ▶); Rizal *et al.* (2008 ▶). For a description of the Cambridge Structural Database, see: Allen (2002 ▶). 
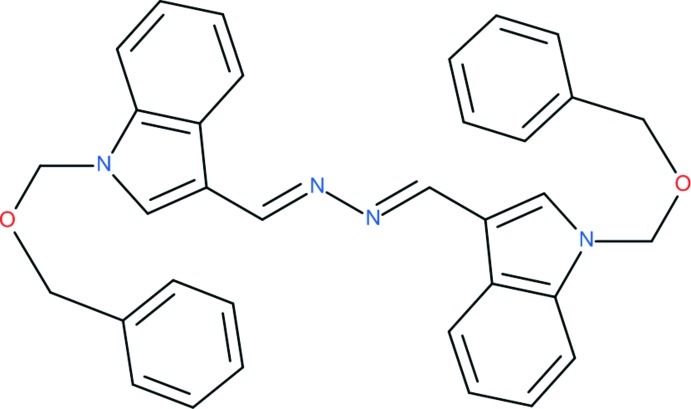


## Experimental

### 

#### Crystal data

C_34_H_30_N_4_O_2_
*M* *_r_* = 526.62
Monoclinic, 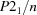

*a* = 12.8518 (10) Å
*b* = 7.9663 (9) Å
*c* = 13.6810 (12) Å
β = 103.672 (5)°
*V* = 1361.0 (2) Å^3^
*Z* = 2
Mo *K*α radiation
μ = 0.08 mm^−1^
*T* = 90 K
0.18 × 0.10 × 0.05 mm

#### Data collection

Nonius KappaCCD diffractometer
Absorption correction: multi-scan (*HKL* *SCALEPACK*; Otwinowski & Minor 1997 ▶) *T*_min_ = 0.986, *T*_max_ = 0.996
4340 measured reflections
2678 independent reflections
1481 reflections with *I* > 2σ(*I*)
*R*_int_ = 0.067

#### Refinement

*R*[*F* ^2^ > 2σ(*F* ^2^)] = 0.056
*wR*(*F* ^2^) = 0.126
*S* = 0.99
2678 reflections
182 parameters
H-atom parameters constrained
Δρ_max_ = 0.25 e Å^−3^
Δρ_min_ = −0.25 e Å^−3^

### 

Data collection: *COLLECT* (Nonius, 2000 ▶); cell refinement: *HKL* *SCALEPACK* (Otwinowski & Minor 1997 ▶); data reduction: *HKL* *DENZO* and *SCALEPACK* (Otwinowski & Minor 1997 ▶); program(s) used to solve structure: *SHELXS86* (Sheldrick, 2008 ▶); program(s) used to refine structure: *SHELXL97* (Sheldrick, 2008 ▶); molecular graphics: *ORTEP-3 for Windows* (Farrugia, 1997 ▶); software used to prepare material for publication: *WinGX* publication routines (Farrugia, 1999 ▶).

## Supplementary Material

Crystal structure: contains datablock(s) global, I. DOI: 10.1107/S1600536812042493/pk2449sup1.cif

Structure factors: contains datablock(s) I. DOI: 10.1107/S1600536812042493/pk2449Isup2.hkl

Supplementary material file. DOI: 10.1107/S1600536812042493/pk2449Isup3.cml

Additional supplementary materials:  crystallographic information; 3D view; checkCIF report

## Figures and Tables

**Fig. 1.**
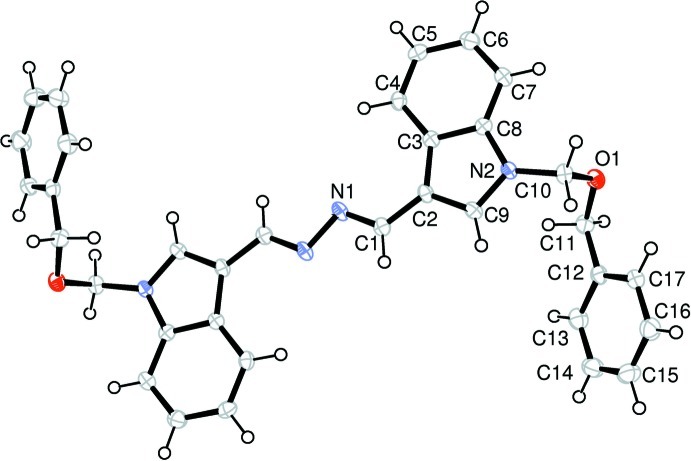
View of (I) (50% probability displacement ellipsoids)

## supplementary crystallographic information

### Comment

Title compound I was synthesized as part of a project targeting the synthesis of tryptophan and other tyrosine derivatives (Shui, 1994). The molecule occupies a crystallographic inversion center and consists of two 3-substituted 1*H*-indole moieties linked by a (1E,2E)-1,2-dimethylenehydrazine bridge, one of numerous examples in which two aromatic ring systems are joined by this 4-atom bridge. The bridge geometry is consistent with that found, for example, in Ph—CH═N—N═CH—Ph [CAS:1048662–10-7, Mom & de With (1978); Burke-Laing & Laing (1976); CSD:BZAZIN02, BZAZIN11, respectively, Allen (2002)]. The geometry of the indole is also consistent with that found in other 1,3-disubstituted indoles [*e.g.*, CSD:OBAVIW, CAS:256391–53-4, Biswas *et al.* (1999)].

### Experimental

The synthesis of I is detailed by Shui (1994), who prepared a suitable single-crystal by recrystallization from dichloromethane.

### Refinement

All H atoms were placed in calculated positions, guided by difference maps, with C—H bond distances 0.95 (aromatic C) and 0.99 (alkyl C) Å, and *U*_iso_=1.2*U*_eq_, thereafter refined as riding.

### Crystal data

**Table:**

C_34_H_30_N_4_O_2_	*F*(000) = 556
*M**_r_* = 526.62	*D*_x_ = 1.285 Mg m^−^^3^
Monoclinic, *P*2_1_/*n*	Mo *K*α radiation, λ = 0.71073 Å
Hall symbol: -P 2yn	Cell parameters from 2472 reflections
*a* = 12.8518 (10) Å	θ = 2.6–26.0°
*b* = 7.9663 (9) Å	µ = 0.08 mm^−^^1^
*c* = 13.6810 (12) Å	*T* = 90 K
β = 103.672 (5)°	Fragment, colorless
*V* = 1361.0 (2) Å^3^	0.18 × 0.10 × 0.05 mm
*Z* = 2	

### Data collection

**Table:**

Nonius KappaCCD diffractometer	2678 independent reflections
Radiation source: sealed tube	1481 reflections with *I* > 2σ(*I*)
Horizonally mounted graphite crystal monochromator	*R*_int_ = 0.067
Detector resolution: 9 pixels mm^-1^	θ_max_ = 26.0°, θ_min_ = 3.0°
CCD rotation images, thick slices scans	*h* = −15→15
Absorption correction: multi-scan (*HKL**SCALEPACK*; Otwinowski & Minor 1997)	*k* = −8→9
*T*_min_ = 0.986, *T*_max_ = 0.996	*l* = −16→16
4340 measured reflections	

### Refinement

**Table:**

Refinement on *F*^2^	Secondary atom site location: difference Fourier map
Least-squares matrix: full	Hydrogen site location: inferred from neighbouring sites
*R*[*F*^2^ > 2σ(*F*^2^)] = 0.056	H-atom parameters constrained
*wR*(*F*^2^) = 0.126	*w* = 1/[σ^2^(*F*_o_^2^) + (0.0483*P*)^2^ + 0.249*P*] where *P* = (*F*_o_^2^ + 2*F*_c_^2^)/3
*S* = 0.99	(Δ/σ)_max_ = 0.001
2678 reflections	Δρ_max_ = 0.25 e Å^−^^3^
182 parameters	Δρ_min_ = −0.25 e Å^−^^3^
0 restraints	Extinction correction: *SHELXL*, Fc^*^=kFc[1+0.001xFc^2^λ^3^/sin(2θ)]^-1/4^
0 constraints	Extinction coefficient: 0.0062 (13)
Primary atom site location: structure-invariant direct methods	

### Special details

**Table:**

Geometry. All e.s.d.'s (except the e.s.d. in the dihedral angle between two l.s. planes) are estimated using the full covariance matrix. The cell e.s.d.'s are taken into account individually in the estimation of e.s.d.'s in distances, angles and torsion angles; correlations between e.s.d.'s in cell parameters are only used when they are defined by crystal symmetry. An approximate (isotropic) treatment of cell e.s.d.'s is used for estimating e.s.d.'s involving l.s. planes.

### Fractional atomic coordinates and isotropic or equivalent isotropic displacement parameters (Å^2^)

**Table:**

	*x*	*y*	*z*	*U*_iso_*/*U*_eq_	
N1	0.02086 (15)	0.0030 (3)	0.55263 (14)	0.0211 (5)	
C1	0.11765 (18)	−0.0511 (3)	0.57786 (17)	0.0206 (6)	
H1	0.15	−0.0917	0.5268	0.025*	
C2	0.17927 (18)	−0.0526 (3)	0.68083 (16)	0.0191 (6)	
C3	0.14858 (18)	−0.0067 (3)	0.77188 (17)	0.0180 (6)	
C4	0.05355 (19)	0.0445 (3)	0.79619 (18)	0.0207 (6)	
H4	−0.0108	0.0538	0.7452	0.025*	
C5	0.05426 (19)	0.0813 (3)	0.89494 (18)	0.0236 (6)	
H5	−0.0098	0.1178	0.9115	0.028*	
C6	0.14857 (19)	0.0654 (3)	0.97152 (19)	0.0258 (7)	
H6	0.147	0.0915	1.0389	0.031*	
C7	0.24299 (19)	0.0127 (3)	0.95068 (17)	0.0226 (6)	
H7	0.3066	0.0011	1.0023	0.027*	
C8	0.24147 (18)	−0.0229 (3)	0.85072 (17)	0.0182 (6)	
N2	0.32454 (15)	−0.0785 (3)	0.81007 (14)	0.0190 (5)	
C9	0.28592 (19)	−0.0961 (3)	0.70805 (17)	0.0205 (6)	
H9	0.3269	−0.133	0.6628	0.025*	
C10	0.43362 (18)	−0.1094 (3)	0.86494 (18)	0.0218 (6)	
H10A	0.4327	−0.1657	0.9293	0.026*	
H10B	0.468	−0.1866	0.8254	0.026*	
O1	0.49589 (12)	0.0398 (2)	0.88566 (11)	0.0225 (4)	
C11	0.50844 (19)	0.1279 (3)	0.79804 (18)	0.0241 (6)	
H11A	0.5435	0.237	0.8194	0.029*	
H11B	0.4365	0.152	0.7552	0.029*	
C12	0.57299 (18)	0.0365 (3)	0.73523 (18)	0.0219 (6)	
C13	0.5658 (2)	0.0890 (3)	0.63721 (18)	0.0276 (7)	
H13	0.5168	0.1754	0.6092	0.033*	
C14	0.6293 (2)	0.0168 (4)	0.57959 (19)	0.0329 (7)	
H14	0.6248	0.0562	0.5132	0.039*	
C15	0.6992 (2)	−0.1122 (4)	0.6180 (2)	0.0352 (7)	
H15	0.7423	−0.1619	0.5782	0.042*	
C16	0.7056 (2)	−0.1679 (4)	0.71520 (19)	0.0328 (7)	
H16	0.7525	−0.2576	0.7419	0.039*	
C17	0.64369 (19)	−0.0929 (3)	0.77379 (19)	0.0263 (7)	
H17	0.6497	−0.1304	0.8408	0.032*	

### Atomic displacement parameters (Å^2^)

**Table:**

	*U*^11^	*U*^22^	*U*^33^	*U*^12^	*U*^13^	*U*^23^
N1	0.0247 (12)	0.0193 (12)	0.0164 (10)	0.0004 (11)	−0.0011 (9)	−0.0027 (10)
C1	0.0221 (14)	0.0174 (15)	0.0212 (13)	−0.0012 (12)	0.0030 (11)	−0.0005 (11)
C2	0.0203 (12)	0.0162 (14)	0.0186 (13)	0.0005 (11)	0.0004 (10)	−0.0009 (11)
C3	0.0209 (12)	0.0141 (14)	0.0185 (13)	−0.0019 (12)	0.0038 (10)	0.0015 (11)
C4	0.0190 (12)	0.0189 (14)	0.0225 (14)	−0.0022 (12)	0.0017 (10)	0.0011 (12)
C5	0.0235 (13)	0.0229 (16)	0.0272 (14)	−0.0017 (12)	0.0116 (11)	0.0002 (12)
C6	0.0292 (15)	0.0261 (17)	0.0228 (14)	−0.0024 (13)	0.0074 (12)	−0.0007 (12)
C7	0.0253 (13)	0.0217 (15)	0.0189 (13)	0.0000 (13)	0.0016 (11)	−0.0007 (12)
C8	0.0196 (12)	0.0136 (14)	0.0218 (13)	−0.0007 (12)	0.0059 (10)	0.0000 (11)
N2	0.0198 (11)	0.0198 (13)	0.0161 (11)	0.0003 (9)	0.0013 (8)	−0.0012 (9)
C9	0.0237 (13)	0.0197 (15)	0.0172 (13)	0.0002 (12)	0.0031 (10)	−0.0017 (11)
C10	0.0214 (13)	0.0179 (15)	0.0242 (14)	0.0003 (12)	0.0016 (11)	0.0000 (12)
O1	0.0240 (9)	0.0201 (10)	0.0220 (9)	−0.0030 (8)	0.0025 (7)	−0.0021 (8)
C11	0.0241 (13)	0.0227 (15)	0.0252 (14)	−0.0006 (12)	0.0052 (11)	0.0048 (12)
C12	0.0209 (13)	0.0204 (14)	0.0224 (13)	−0.0035 (12)	0.0016 (11)	−0.0009 (12)
C13	0.0292 (14)	0.0246 (16)	0.0274 (15)	−0.0002 (13)	0.0031 (12)	0.0045 (13)
C14	0.0357 (15)	0.0386 (19)	0.0251 (15)	−0.0055 (15)	0.0088 (12)	−0.0007 (14)
C15	0.0326 (16)	0.0410 (19)	0.0348 (17)	0.0004 (15)	0.0136 (13)	−0.0079 (15)
C16	0.0295 (15)	0.0346 (18)	0.0324 (16)	0.0066 (14)	0.0036 (12)	−0.0025 (14)
C17	0.0229 (14)	0.0330 (17)	0.0218 (14)	0.0017 (13)	0.0030 (11)	−0.0008 (13)

### Geometric parameters (Å, º)

**Table:**

N1—C1	1.284 (3)	C9—H9	0.95
N1—N1^i^	1.414 (4)	C10—O1	1.424 (3)
C1—C2	1.444 (3)	C10—H10A	0.99
C1—H1	0.95	C10—H10B	0.99
C2—C9	1.377 (3)	O1—C11	1.431 (3)
C2—C3	1.440 (3)	C11—C12	1.514 (3)
C3—C4	1.400 (3)	C11—H11A	0.99
C3—C8	1.413 (3)	C11—H11B	0.99
C4—C5	1.380 (3)	C12—C13	1.387 (3)
C4—H4	0.95	C12—C17	1.393 (4)
C5—C6	1.408 (3)	C13—C14	1.386 (3)
C5—H5	0.95	C13—H13	0.95
C6—C7	1.376 (3)	C14—C15	1.384 (4)
C6—H6	0.95	C14—H14	0.95
C7—C8	1.392 (3)	C15—C16	1.385 (4)
C7—H7	0.95	C15—H15	0.95
C8—N2	1.388 (3)	C16—C17	1.391 (3)
N2—C9	1.373 (3)	C16—H16	0.95
N2—C10	1.446 (3)	C17—H17	0.95
			
C1—N1—N1^i^	111.9 (2)	O1—C10—N2	113.05 (19)
N1—C1—C2	122.6 (2)	O1—C10—H10A	109
N1—C1—H1	118.7	N2—C10—H10A	109
C2—C1—H1	118.7	O1—C10—H10B	109
C9—C2—C3	106.77 (19)	N2—C10—H10B	109
C9—C2—C1	123.2 (2)	H10A—C10—H10B	107.8
C3—C2—C1	130.0 (2)	C10—O1—C11	114.34 (17)
C4—C3—C8	118.2 (2)	O1—C11—C12	115.2 (2)
C4—C3—C2	135.4 (2)	O1—C11—H11A	108.5
C8—C3—C2	106.41 (19)	C12—C11—H11A	108.5
C5—C4—C3	119.3 (2)	O1—C11—H11B	108.5
C5—C4—H4	120.3	C12—C11—H11B	108.5
C3—C4—H4	120.3	H11A—C11—H11B	107.5
C4—C5—C6	121.0 (2)	C13—C12—C17	118.5 (2)
C4—C5—H5	119.5	C13—C12—C11	118.9 (2)
C6—C5—H5	119.5	C17—C12—C11	122.5 (2)
C7—C6—C5	121.3 (2)	C12—C13—C14	120.8 (2)
C7—C6—H6	119.4	C12—C13—H13	119.6
C5—C6—H6	119.4	C14—C13—H13	119.6
C6—C7—C8	117.2 (2)	C15—C14—C13	120.5 (2)
C6—C7—H7	121.4	C15—C14—H14	119.7
C8—C7—H7	121.4	C13—C14—H14	119.7
N2—C8—C7	128.7 (2)	C16—C15—C14	119.3 (3)
N2—C8—C3	108.3 (2)	C16—C15—H15	120.4
C7—C8—C3	123.0 (2)	C14—C15—H15	120.4
C9—N2—C8	108.20 (19)	C15—C16—C17	120.2 (3)
C9—N2—C10	125.7 (2)	C15—C16—H16	119.9
C8—N2—C10	126.11 (19)	C17—C16—H16	119.9
N2—C9—C2	110.3 (2)	C16—C17—C12	120.7 (2)
N2—C9—H9	124.9	C16—C17—H17	119.6
C2—C9—H9	124.9	C12—C17—H17	119.6
			
C1^i^—N1^i^—N1—C1	180	C7—C8—N2—C10	−1.0 (4)
N1^i^—N1—C1—C2	177.9 (2)	C3—C8—N2—C10	179.2 (2)
N1—C1—C2—C9	−174.1 (2)	C8—N2—C9—C2	0.2 (3)
N1—C1—C2—C3	3.4 (4)	C10—N2—C9—C2	−178.6 (2)
C9—C2—C3—C4	−178.3 (3)	C3—C2—C9—N2	−0.8 (3)
C1—C2—C3—C4	4.0 (5)	C1—C2—C9—N2	177.2 (2)
C9—C2—C3—C8	1.0 (3)	C9—N2—C10—O1	98.5 (3)
C1—C2—C3—C8	−176.8 (2)	C8—N2—C10—O1	−80.1 (3)
C8—C3—C4—C5	1.6 (4)	N2—C10—O1—C11	−62.1 (3)
C2—C3—C4—C5	−179.2 (3)	C10—O1—C11—C12	−66.3 (2)
C3—C4—C5—C6	−1.0 (4)	O1—C11—C12—C13	163.0 (2)
C4—C5—C6—C7	0.0 (4)	O1—C11—C12—C17	−20.4 (3)
C5—C6—C7—C8	0.5 (4)	C17—C12—C13—C14	−1.4 (4)
C6—C7—C8—N2	−179.5 (2)	C11—C12—C13—C14	175.3 (2)
C6—C7—C8—C3	0.2 (4)	C12—C13—C14—C15	1.7 (4)
C4—C3—C8—N2	178.5 (2)	C13—C14—C15—C16	−0.4 (4)
C2—C3—C8—N2	−0.9 (3)	C14—C15—C16—C17	−1.0 (4)
C4—C3—C8—C7	−1.2 (4)	C15—C16—C17—C12	1.3 (4)
C2—C3—C8—C7	179.4 (2)	C13—C12—C17—C16	0.0 (4)
C7—C8—N2—C9	−179.8 (3)	C11—C12—C17—C16	−176.7 (2)
C3—C8—N2—C9	0.4 (3)		

Symmetry code: (i) −*x*, −*y*, −*z*+1.
